# Barriers to Lead Psychiatric Clinical Supervision – A Cross-Sectional Survey

**DOI:** 10.1192/bjo.2025.10414

**Published:** 2025-06-20

**Authors:** Zaib un Nisa, Zong Lai, Kehinde Junaid, Bala Ganesan, Sudheer Lankappa

**Affiliations:** 1 Derbyshire Healthcare NHS Foundation Trust, Derby, United Kingdom; 2 Nottinghamshire Healthcare NHS Foundation Trust, Nottingham, United Kingdom

## Abstract

**Aims:** The Royal College of Psychiatrists (RCPsych) recommends that psychiatric trainees receive one hour of 1:1 supervision per week, with clinical supervisors allocated 0.25 PA (programmed activity) protected time per trainee weekly. The GMC National Training Survey 2023 found that 86% of trainees reported positive feedback on clinical supervision, though the survey was not psychiatry specific. Locally, the Resident Doctors Forum raised concerns about some trainees not receiving the recommended supervision time, prompting the introduction of a new supervision form.

Aims were to identify and assess barriers to providing regular supervision to support the professional development of psychiatrists in training within Nottinghamshire Healthcare NHS Foundation Trust.

**Methods:** A questionnaire was developed based on the “Enablers and Barriers to Effective Clinical Supervision in the Workplace: A Rapid Evidence Review” to identify barriers to effective clinical supervision. It was emailed to all lead clinical supervisors in Adult Mental Health, with a two-week response deadline. The feedback was analysed using a mixed methods approach, combining quantitative and qualitative analysis.

**Results:** The survey received a 30% response rate (21 out of 70 eligible trainers), with a distribution reflecting the grades of resident doctors in the trust: 34% supervising HST, 34% supervising CT, 19% supervising FY, and 13% supervising GPVTS.

Key findings include: 67% of trainers felt their clinical workload allowed sufficient time for supervision, but 81% sometimes had to cancel due to clinical commitments. Trainers with sufficient time for supervision typically had protected time formally agreed in their job plans (85%).

80% of trainers faced cancellations due to trainee unavailability (e.g., shift work, staff shortages), and 10% felt supervision was hindered by inadequate resources, such as lack of private spaces.

Awareness of the RCPsych supervision guidance was low (33%), and 50% were not familiar with or did not use the local supervision form. Opinions on the form were divided: half found it helpful, while the other half saw it as additional workload.

Major barriers to effective supervision included intense clinical workload, time pressure, staff shortages, managing multiple trainees, and trainee unavailability due to on-call or leave commitments.

**Conclusion:** Suggested actions to address these barriers include:

Distributing the RCPsych guidance and Supervision Form to all trainers.

Encouraging supervisors to schedule supervision mid-week to avoid conflicts with on-call shifts.

Supervisors should discuss protected time in their job plans with clinical directors and work with medical education to find private workspaces for supervision.

